# Associations of COVID-19 vaccination during pregnancy with adverse neonatal and maternal outcomes: A systematic review and meta-analysis

**DOI:** 10.3389/fpubh.2023.1044031

**Published:** 2023-01-30

**Authors:** Cailin Ding, Yakun Liu, Wenbo Pang, Dan Zhang, Kai Wang, Yajun Chen

**Affiliations:** Department of General Surgery, Beijing Children's Hospital, Capital Medical University, National Center for Children's Health, Beijing, China

**Keywords:** COVID-19, vaccination, pregnancy, adverse outcomes, neonatal, maternal

## Abstract

**Objectives:**

The low COVID-19 vaccine uptake rate among pregnant women is mainly due to safety concerns about COVID-19 vaccines due to limited safety evidence. Our goal was to evaluate the safety of COVID-19 vaccination during pregnancy with up-to-date evidence.

**Methods:**

A comprehensive search of MEDLINE, EMBASE, the Cochrane Library, and clinicaltrials.gov was performed on April 5th, 2022, and updated on May 25th, 2022. Studies evaluating the association of COVID-19 vaccination during pregnancy with adverse maternal and neonatal outcomes were included. Two reviewers independently performed the risk of bias assessment and data extraction. Inverse variance random effect meta-analyses were performed to pool outcome data.

**Results:**

Forty-three observational studies were included. COVID-19 vaccination [96,384 (73.9%) BNT162b2, 30,889 (23.7%) mRNA-1273, and 3,172 (2.4%) other types] during pregnancy [23,721 (18.3%) in the first trimester, 52,778 (40.5%) in the second trimester, and 53,886 (41.2%) in the third trimester].was associated with reduced risks of stillbirth or neonatal death (OR, 0.74; 95% CI, 0.60–0.92). Sensitivity analysis restricted to studies in participants without COVID-19 showed that the pooled effect was not robust. COVID-19 vaccination during pregnancy was not associated with congenital anomalies (OR, 0.83; 95% CI, 0.63–1.08), preterm birth (OR, 0.98; 95% CI, 0.90–1.06), NICU admission or hospitalization (OR, 0.94; 95% CI, 0.84–1.04), an Apgar score at 5 min <7 (OR, 0.93; 95% CI, 0.86–1.01), low birth weight (OR, 1.00; 95% CI, 0.88–1.14), miscarriage (OR, 0.99; 95% CI, 0.88–1.11), cesarean delivery (OR, 1.07; 95% CI, 0.96–1.19), or postpartum hemorrhage (OR, 0.91; 95% CI, 0.81–1.01).

**Conclusions:**

COVID-19 vaccination during pregnancy was not associated with any of the adverse neonatal or maternal outcomes studied. Interpretation of study findings is limited by the types and timing of vaccination. The vaccinations in our study received during pregnancy were primarily mRNA vaccines administered in the second and third trimester. Future RCTs and meta-analysis are warranted to evaluate the efficacy and long-term effects of the COVID-19 vaccines.

**Systematic review registration:**

https://www.crd.york.ac.uk/prospero/display_record.php?ID=CRD42022322525, identifier: PROSPERO, CRD42022322525.

## Introduction

Severe acute respiratory syndrome coronavirus-2 (SARS-CoV-2) infection during pregnancy is associated with an increased risk of maternal mortality and severe morbidity (1–3). During pregnancy, alterations in immunological and physiological responses increase a person's susceptibility to SARS-CoV-2 and other viral infections, indicating the need for robust coronavirus disease 2019 (COVID-19) prevention and treatment strategies (4–6).

COVID-19 vaccines have been effective in preventing COVID-19 and lowering the risk of severe infections in the general and pregnant populations, even during the Delta- and Omicron-predominant periods (7–9). Despite international recommendations (10), COVID-19 vaccine acceptance among pregnant people is much lower than that in the general population (11, 12). The main reason for the low rate of COVID-19 vaccine uptake is safety concerns about COVID-19 vaccines regarding both mothers and babies, but these concerns are supported by only limited data (13, 14).

During the early stage of the pandemic, pregnant individuals were frequently excluded from clinical trials of vaccines (15), which led to knowledge gaps regarding the safety and effectiveness of COVID-19 vaccines. Since the authorization of COVID-19 vaccination in pregnant people, studies on the association between vaccination and adverse birth and maternal outcomes have emerged. Increasing evidence supports the safety of COVID-19 vaccination in the pregnant population (12, 16–37). However, most conclusions are limited by small sample sizes, restricted generalizability or unbalanced confounding factors. Hence, we conducted a systematic review and meta-analysis to synthesize available evidence, with the aim of assessing the safety of COVID-19 vaccination during pregnancy and, ultimately, informing pregnant people and health care workers of our findings.

## Methods

This meta-analysis was conducted following the Preferred Reporting Items for Systematic Reviews and Meta-analyses (PRISMA) guidelines. It was previously registered in the International Prospective Register of Systematic Reviews (PROSPERO; Identifier: CRD42022322525).

### Search strategy

Two independent investigators searched MEDLINE, EMBASE, and the Cochrane Library from their inception through April 5th, 2021. In addition, we searched clinicaltrials.gov for ongoing and completed clinical trials. The reference lists of all the included articles were manually searched to identify additional candidate studies. No restrictions were imposed. The detailed search strategy is presented in Supplementary Methods S1.

An updated search was performed on May 25th, 2022.

### Study eligibility and selection

Studies were included if they evaluated the association of maternal COVID-19 vaccination with the primary outcome (stillbirth or neonatal death) or secondary outcomes (miscarriage, congenital anomalies, preterm birth, small for gestational age (SGA), low birth weight, 5-min APGAR score of < 7, neonatal intensive care unit (NICU) admission or hospitalization, gestational weeks at delivery, birth weight, cesarean delivery, postpartum hemorrhage, chorioamnionitis, maternal ICU admission). Studies were excluded if they (1) were not peer-reviewed, (2) included an intervention or control group that received the first dose of the COVID-19 vaccine before conception, (3) were case–control studies with populations grouped by SARS-CoV-2 infection status, (4) included participants from another included article with a larger sample size and identical outcomes of interest, or (5) were case reports.

Two authors independently reviewed all the abstracts and determined if the studies met the inclusion criteria. Full texts were reviewed to determine if the studies fulfilled the exclusion criteria. Discrepancies were resolved *via* consensus or involvement of a third author if disagreements could not be resolved by discussion. Articles reported as only abstracts were included if sufficient information was available to extract outcome data.

### Data extraction

Two researchers independently extracted data from all the included studies using a predetermined data sheet. The following data were extracted from each study: author name, year of publication, geographical region, sample size, characteristics of the participants, vaccination timing, vaccine type, and outcomes of interest. The numbers of observed events and the total numbers of participants at risk were extracted. Means and standard deviations (SDs) were extracted or converted from other statistics for continuous outcomes (e.g., birth weight, gestational week). The collected data were compared between reviewers, and any discrepancies were resolved by discussion or by involving a senior author if disagreements could not be resolved. The authors were contacted by e-mail to obtain potential missing data of interest. Studies were identified by geographical region, database, time of vaccination, and time of delivery. The articles from the same population were reviewed to exclude duplicated outcome data.

### Quality assessment

The methodological quality of the evidence and risk of bias were evaluated by two independent authors using the Newcastle–Ottawa Scale (38). The total scores ranged from 0 (worst) to 9 (best) for cohort or case–control studies. We considered a study to have a low risk of bias if the total score was at least 7 and a high risk of bias if the score was 6 or lower. Any disagreements were resolved by consensus *via* face-to-face discussion.

### Data synthesis and analysis

We conducted a random-effects meta-analysis using RevMan software (version 5.4; Cochrane Collaboration). Pooled odds ratios (ORs) and 95% confidence intervals (CIs) were generated for dichotomous outcomes. Mean differences (MDs) and 95% CIs were calculated for continuous outcomes.

Statistical heterogeneity was assessed using the *I*^2^ statistic. Heterogeneity was considered significant if the *I*^2^ value was >50%. Publication bias was assessed by visually inspecting funnel plots for results comprising seven or more studies. *Post hoc* subgroup analysis by the World Health Organization (WHO) geographic region and source of outcome data (medical records or registry or survey) was conducted for results with significant heterogeneity (*I*^2^ > 50%). Prespecified subgroup analysis could not be conducted in accordance with previous registration (PROSPERO; Identifier: CRD42022322525), because sufficient data could not be obtained.

### Sensitivity analysis

To evaluate the robustness of the pooled results, we conducted sensitivity analyses for the following: (1) vaccination during the first trimester, as the first trimester is generally considered the most vulnerable period for the fetus in the context of medication, infectious agent, and toxin exposure (39); (2) studies with adjusted results or matched controls, as vaccinated and unvaccinated pregnant individuals tend to have inconsistent characteristics (40); (3) high quality studies; (4) participants who did not contract COVID-19 after vaccination, as SARS-CoV-2 infection is associated with increased maternal and neonatal morbidity (2, 41).

## Results

**Figure 1:**
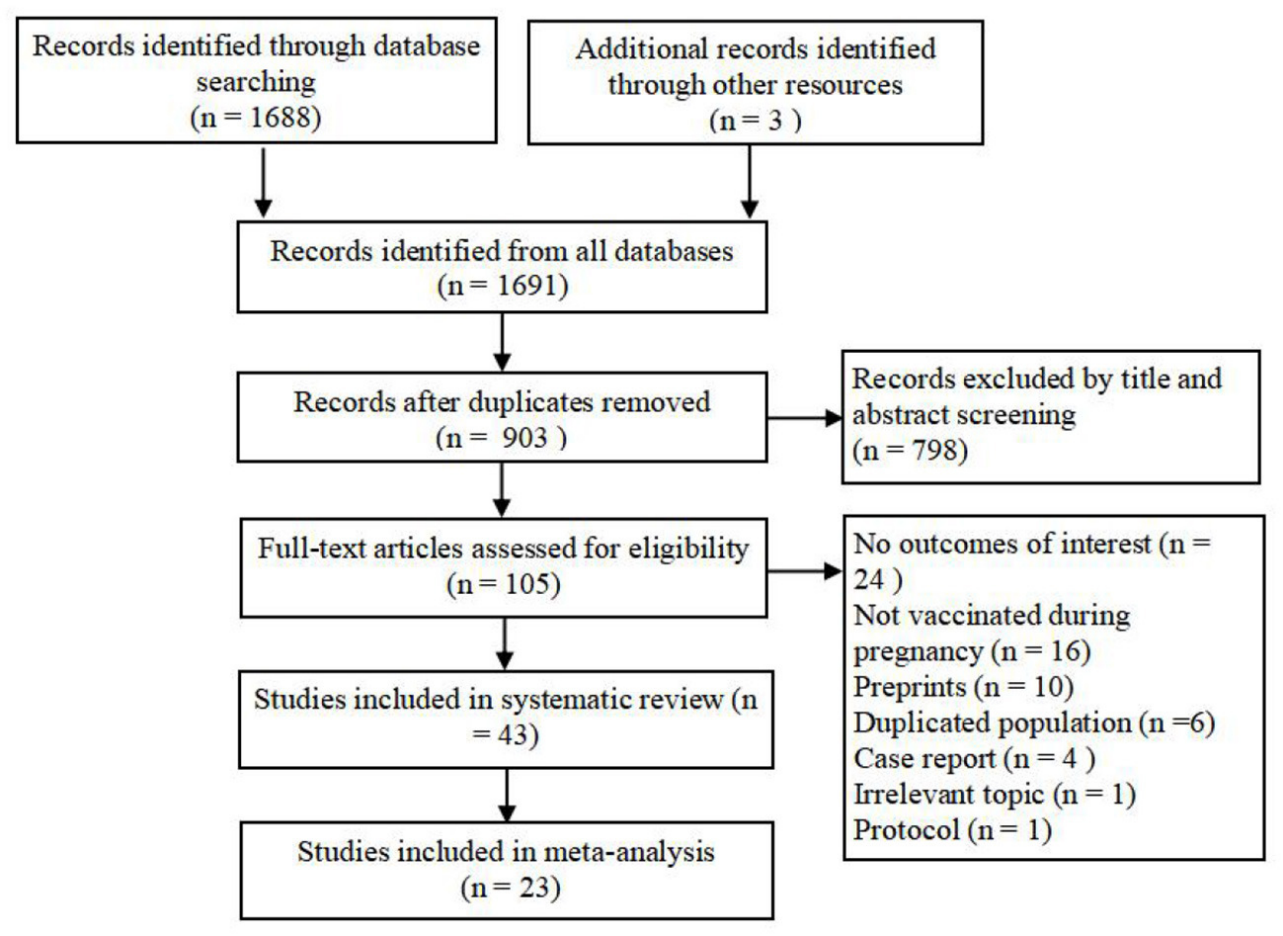
Study selection process.

A total of 1,688 references were obtained from the initial and undated electronic database search, and three were obtained from a manual search of citations. After eligibility screening, 43 observational studies were included in the systematic review and 23 in the final meta-analysis (Figure 1) (12, 16–37).

In total, 599,956 pregnant individuals were included in the 43 studies. There were 24 studies with 369,397 pregnant people from the European region, 17 studies with 229,576 pregnant people from the American region, and the remaining two studies were from the West Pacific region. Of the 130,445 pregnant individuals who received vaccines, 96,384 (73.9%) received BNT162b2, 30,889 (23.7%) received mRNA-1273, and 3,172 (2.4%) received other types. Of the 130,385 pregnant people with an identified time of vaccination, 23,721 (18.3%) were vaccinated in the first trimester, 52,778 (40.5%) in the second trimester, and 53,886 (41.2%) in the third trimester.

Twenty-three cohort or case–control studies were included for data synthesis, and quality assessment was performed for these studies (12, 16–37). Eighteen studies (78%) were labeled as having a low risk of bias (12, 16, 17, 19–24, 26–31, 33, 34, 37). The main source of bias was “comparability,” which was required for adjustments for age and SARS-CoV-2 infection.

The vaccine type, timing, and outcomes of interest and quality assessments are detailed in Supplementary Tables S1–S4.

### Primary outcome

**Figure 2:**
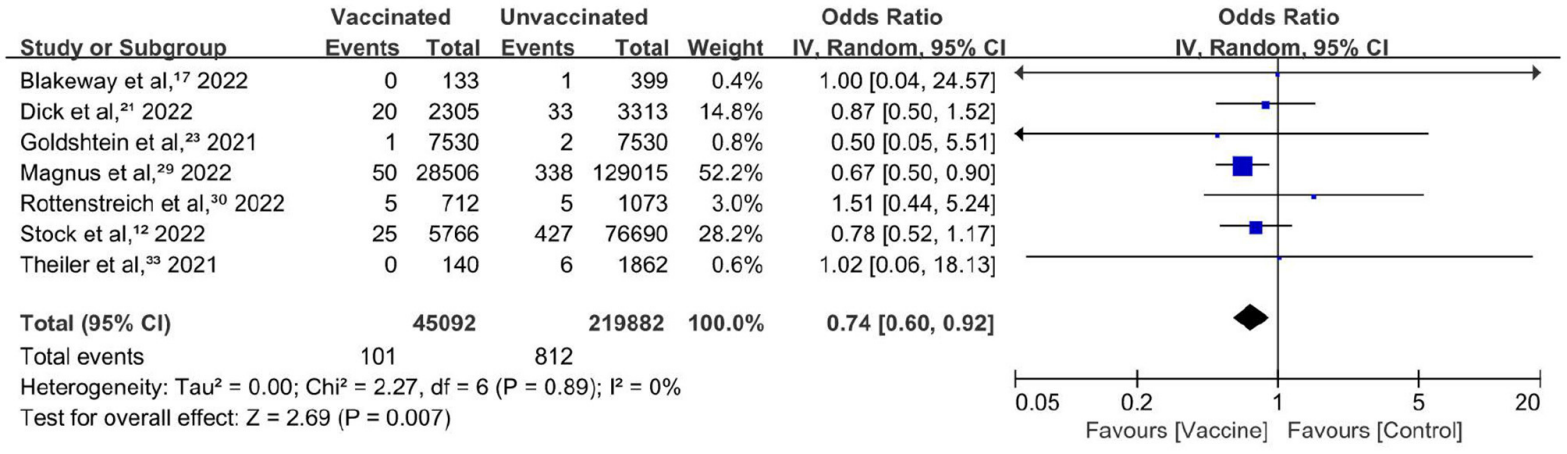
Forest plot for stillbirth or neonatal death.

Ten studies reported the incidence of stillbirth or neonatal death. The incidence ranged from 0 to 0.9% in vaccinated pregnant people. Included in the meta-analysis were seven cohort studies involving 26,497 participants (12, 17, 21, 23, 29, 30, 33). All these studies (100%) were of high quality. Overall, COVID-19 vaccination during pregnancy was associated with a reduced risk of stillbirth or neonatal death within 28 days after birth (Figure 2), with a pooled OR of 0.74 (95% CI, 0.60–0.92; *P* = 0.007; *I*^2^ = 0%). A statistically significant level was reached in one study with 52.2% weighting. Three studies had wide ranges of 95% CIs, and the weightings were much lower. The heterogeneity was very low.

Sensitivity analysis restricted to studies comprising participants who did not contract COVID-19 after vaccination (four studies; OR, 0.95; 95% CI, 0.58–1.56; *P* = 0.85; *I*^2^ = 0%) or studies that adjusted for baseline characteristics or other potential factors (three studies; OR, 0.77; 95% CI, 0.42–1.38; *P* = 0.37; *I*^2^ = 0%) showed that the pooled effect was not robust (Supplementary Tables S5, S6).

### Secondary outcomes

Congenital anomalies were identified by prenatal ultrasonography or after birth. In the meta-analysis, four studies included 4,782 vaccinated pregnant persons (17, 18, 24, 31). Overall, COVID-19 vaccination during pregnancy was not associated with fetal or neonatal congenital anomalies (OR, 0.83; 95% CI, 0.63–1.08; *P* = 0.16; *I*^2^ = 0%). Among pregnant people vaccinated in the first trimester (two studies, 2,872 vaccinated individuals), the pooled risk did not increase (OR, 0.73; 95% CI, 0.52–1.01; *P* = 0.06; *I*^2^ = 0%). Sensitivity analysis limited to studies with matched controls or adjusted results (two studies; OR, 0.83; 95% CI, 0.58–1.19; *P* = 0.31; *I*^2^ = 0%) and studies in which all participants did not contract COVID-19 after vaccination (one study; OR, 0.81; 95% CI, 0.22–2.96; *P* = 0.75) did not show significant differences. The results were presented in Supplementary Figure S1 and Supplementary Tables S5–S8.

**Figure 3:**
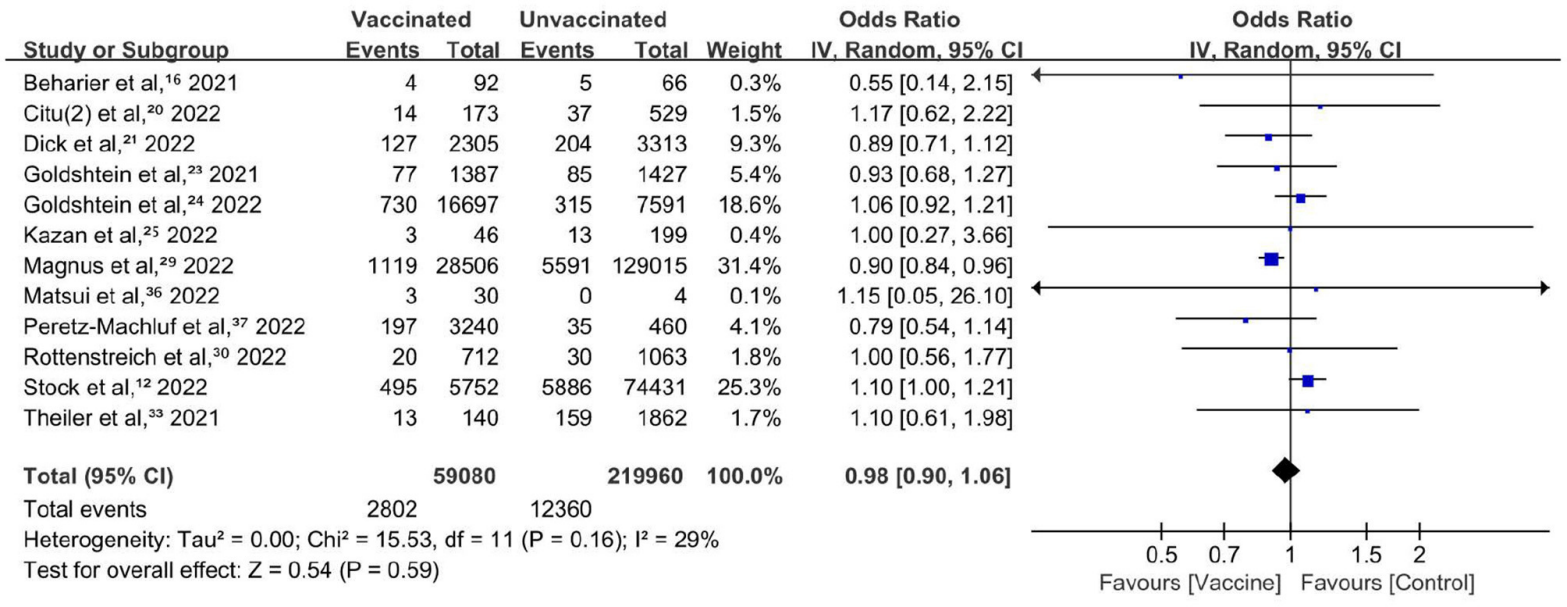
Forest plot for preterm birth.

Preterm birth was reported in 13 studies with 59,080 vaccinated and 219,960 unvaccinated individuals (12, 18, 20, 21, 23–25, 27, 29, 30, 33, 36, 37). There was no significant difference between the vaccinated and unvaccinated groups (OR, 0.98; 95% CI, 0.90–1.06; *P* = 0.59; *I*^2^ = 29%; Figure 3). Another study compared risk using adjusted hazard ratios and did not show a significant difference (0.91, 95% CI: 0.82–1.01) between vaccinated and unvaccinated pregnant persons (27). Sensitivity analysis restricted to studies with participants who did not contract COVID-19 after vaccination (six studies; OR, 0.88; 95% CI, 0.78–0.99; *P* = 0.03; *I*^2^ = 0%) and studies with adjustment or baseline matching (five studies; OR, 0.88; 95% CI, 0.80–0.98; *P* = 0.02; *I*^2^ = 0%) revealed a decreased risk of preterm birth.

SGA was reported in eight studies with 51,846 vaccinated and 145,237 unvaccinated pregnant people (17, 20, 21, 24, 29, 30, 34, 37). The pooled OR was 0.95 (95% CI, 0.86–1.04, *P* = 0.24, *I*^2^ = 39%; Supplementary Figure S2). However, the result was not stable in any of the prespecified sensitivity analyses. Another study compared risk using adjusted hazard ratios and did not show a significant difference (0.95, 95% CI: 0.87–1.03) (27).

NICU admission or hospitalization was reported in eight studies with 72,266 vaccinated and 170,565 unvaccinated pregnant individuals (16, 17, 22, 24, 29, 30, 33, 37). Overall, COVID-19 vaccination during pregnancy was not associated with NICU admission or hospitalization after birth (OR, 0.94; 95% CI, 0.84–1.04; *P* = 0.22; *I*^2^ = 70%; Supplementary Figure S3). However, significant heterogeneity was detected. Subgroup analysis by geographical region addressed the heterogeneity and showed a decreased risk of NICU admission or hospitalization in the Americas (three studies; OR, 0.84; 95% CI, 0.80–0.89; *P* < 0.001; *I*^2^ = 0%; Supplementary Table S9). Sensitivity analysis restricted to studies with matched controls or adjusted results also showed a protective effect of COVID-19 vaccination (six studies; OR, 0.93; 95% CI, 0.89–0.98; *P* = 0.003; *I*^2^ = 0%), while other sensitivity analyses did not.

COVID-19 vaccination during pregnancy was not associated with an Apgar score < 7 at 5 min (20–22, 29, 30, 33, 34, 37) (eight studies; OR, 0.93; 95% CI, 0.86–1.01; *P* = 0.07, *I*^2^ = 0%) or low birth weight (24, 33) (two studies; OR, 1.00; 95% CI, 0.88–1.14; *P* = 0.98, *I*^2^ = 0%). COVID-19 vaccination during pregnancy had no effects on birthweight (16, 20–23, 30, 34, 35, 37) (nine studies; MD, 0.81 g; 95% CI, −15.55–17.18 g; *P* = 0.92, *I*^2^ = 59%) or gestational age at delivery (16, 21–23, 30, 32, 34–37) (10 studies; MD, −0.05 week; 95% CI, −0.11–0.01 week; *P* = 0.10, *I*^2^ = 63%). The results are depicted in Supplementary Figures S4–S7. The subgroup analysis of birth weight and gestational age at delivery are presented in Supplementary Table S9, S10.

The risk of miscarriage was reported in 4 studies with 9,662 vaccinated and 27,295 unvaccinated pregnant persons (18, 19, 23, 28). The overall OR was 0.99 (95% CI, 0.88–1.11; *P* = 0.85; *I*^2^ = 0%; Supplementary Figure S8). Two studies reported results from pregnant people vaccinated during the first trimester (19, 28), with a pooled OR of 1.03 (95% CI, 0.89–1.20; *P* = 0.65; *I*^2^ = 0%).

Maternal COVID-19 vaccination during pregnancy was not associated with an increased risk of cesarean delivery (17, 20–22, 30, 33, 34, 37) (eight studies; OR, 1.07; 95% CI, 0.96–1.19; *P* = 0.25, *I*^2^ = 59%; Supplementary Figure S9). When the analysis was restricted to the study population without COVID-19, the risk was similar (six studies; OR, 1.09; 95% CI, 0.92–1.28; *P* = 0.33, *I*^2^ = 55%). In the analysis restricted to studies controlled for potential confounders, the risk was significantly decreased to a statistically significant level (four studies; OR, 0.96; 95% CI, 0.93–0.99; *P* = 0.006, *I*^2^ = 0%). The subgroup analysis of cesarean delivery are presented in Supplementary Table S11.

The risk of postpartum hemorrhage was close to the significance threshold (17, 20–22, 30, 33, 34) (seven studies; OR, 0.91; 95% CI, 0.81–1.01; *P* = 0.08, *I*^2^ = 5%). The risks of chorioamnionitis (22, 30), placental abruption (17, 20, 30, 34), and maternal ICU admission (17, 30, 33) were reported less frequently. Their associations with prenatal COVID-19 vaccination were not statistically significant. The detailed results are presented in Table 1 and Supplementary Figures S10–S13.

**Table 1:** Pooled results of association of COVID-19 vaccination during pregnancy and adverse birth outcomes.

	**Included studies**	**Participants**	**Effect size (OR)**	**95% CI**	***P*-value**	***I* ^2^**	**False-*N***
Stillbirth or neonatal death	7	264,974	0.74	0.60–0.92	0.007	0	0
Congenital anomalies	4	9,423	0.83	0.63–1.08	0.16	0	0
Preterm birth	12	279,040	0.98	0.90–1.06	0.59	29	0
Small for gestational age	8	197,083	0.95	0.86–1.04	0.24	39	1
NICU admission or hospitalization	8	242,831	0.94	0.84–1.04	0.22	70	4
Apgar (5 min) < 7	7	227,639	0.93	0.86–1.01	0.07	0	0
Low birth weight	2	24,838	1.00	0.88–1.14	0.98	0	–
Miscarriage	4	36,957	0.99	0.88–1.11	0.85	0	0
Postpartum hemorrhage	7	67,803	0.91	0.81–1.01	0.08	5	0
Cesarean delivery	8	71,947	1.07	0.96–1.19	0.25	59	9
Chorioamnionitis	2	54,550	1.18	0.65–2.13	0.59	66	–
Maternal ICU admission	2	2,534	2.05	0.65–6.50	0.22	22	–
Placental abruption	3	6,876	0.66	0.38–1.16	0.15	0	0
Birthweight	9	84,210	MD, 0.81 g	MD, −15.55 to 17.18 g	0.92	59	0
Gestational age at delivery	10	71,496	MD, −0.05 w	MD, −0.11 to 0.01 w	0.10	63	0

### Sensitivity analysis and publication bias

The predetermined sensitivity analyses are detailed in Supplementary Tables S9–S12. Publication bias was assessed by visually inspecting funnel plots. No asymmetry was found except for stillbirth or neonatal death, an Apgar score < 7 at 5 min, and postpartum hemorrhage. The possible sources of asymmetry in funnel plots may be possibly related to the small sample size. No publication bias was found.

### Ongoing studies

To date, no RCTs are available for analysis. However, 13 candidate clinical trials are active (Clinicaltrails.gov: NCT04659759, NCT04705116, NCT04754594, NCT04765384, NCT04826640, NCT04957953, NCT04958304, NCT05031468, NCT05115617, NCT05197621; International Clinical Trials Registry Platform: EUCTR2020-005444-35-ES, EUCTR2021-002327-38-NL, ISRCTN15279830).

## Discussion

This meta-analysis showed that COVID-19 vaccination during pregnancy was associated with overall reduced risks of stillbirth or newborn death. COVID-19 vaccination during pregnancy was not associated with the adverse neonatal outcomes or adverse maternal outcomes of interest. Although several results were not robust in the sensitivity analyses, no increased risks of any adverse birth outcomes were detected in vaccinated pregnant people.

Approximately 210 million pregnancies occur each year, indicating that pregnant people are not a marginal population (42). SARS-CoV-2 infection in pregnancy is associated with several severe consequences (1, 2), highlighting the need for prevention measures against SARS-CoV-2 infection. COVID-19 vaccines have been shown to be highly effective against SARS-CoV-2 infection, severe COVID-19, and death in clinical trials and in real-world settings (7, 43). However, despite cohort studies and ongoing clinical trials in this population (44, 45), evidence of their safety and efficacy are limited to date, which contributes to immunization hesitancy (14, 46). Hence, it is beneficial to synthesize all available data to evaluate safety. These data are particularly important for improvement of COVID-19 vaccine uptake in pregnant people (47, 48).

One of our primary findings was a decreased risk of stillbirth or newborn death. The inherent nature of vaccination may not promote the survival of fetuses and newborns (49). It is speculated that the effect may be attributed to the function of abating the severity of or preventing infection. Hence, we conducted a sensitivity analysis by excluding the influence of SARS-CoV-2 infection, which resulted in a null effect and verified the hypothesis.

COVID-19 vaccination during pregnancy showed protective effects against preterm birth, NICU admission or hospitalization, and cesarean delivery in the sensitivity analysis. However, the number of included studies was relatively small.

The first trimester is the period with the highest risk of teratogenic effects on fetal development (39). Teratogen exposure in this critical period could disrupt embryogenesis and increase the risk for organic or systematic abnormalities. Hence, potential teratogenic risk should be evaluated among pregnant people vaccinated in the early stage of pregnancy. The pooled results of 6,976 pregnant individuals vaccinated in the first trimester did not detect any teratogenic effects caused by current COVID-19 vaccines. However, the number of studies included in the quantitative synthesis was very limited.

Potential maternal safety issues are also cited as a primary reason for reluctance to receive the COVID-19 vaccine among the pregnant population (13, 46). Several common adverse maternal outcomes were assessed in this study, and no association with COVID-19 vaccination was identified.

This study has several limitations. First, the findings of this study were based on observational studies, most of which were cohort studies. Consequently, the level of evidence of the current meta-analysis is limited. Second, in most studies, the baseline characteristics were not balanced. Vaccinated and unvaccinated individuals tended to have inconsistent demographic features, which may have introduced substantial selection bias. Although some studies provided adjusted results, the inherent pitfalls of cohort studies do not allow for strict control of potential confounders. Third, for the sensitivity analyses, the number of studies for each outcome was limited, although some studies included a very large number of pregnant people. Fourth, there could have been an overlap of participants identified from the birth or vaccination registry database with those from hospital medical records. Fifth, more than 90% of the COVID-19 vaccines were from four manufacturers (Pfizer, Modena, Janssen, and AstraZeneca), and most participants received a mRNA vaccine. The first vaccines authorized by governments were produced by the aforementioned manufacturers; as a result, the generalizability of our findings to pregnant individuals who received a different vaccine is limited. Sixth, first trimester vaccine exposure may be critical in evaluating the potential risks of miscarriage and congenital anomalies. However, the majority of individuals had been vaccinated after the period of greatest risk. The increased risk of COVID-19 persists throughout pregnancy, and the uptake of vaccines during the early period offers persistent protection against COVID-19 during pregnancy. Seventh, all the included studies were from European and American regions, and the initial recommendations for vaccination during pregnancy were for health care workers and individuals with underlying health conditions (50). Despite a diversity of ethnicities, generalizability is still limited at the worldwide level. Eighth, we could not evaluate the long-term adverse effects of COVID-19 vaccines, as COVID-19 vaccination during pregnancy was not officially offered until the end of 2021, and data from initial clinical trials are scarce. Since observational studies are emerging and dozens of RCTs are ongoing, we will continuously surveil and update this systematic review and meta-analysis when relevant new evidence becomes available.

## Conclusions

COVID-19 vaccination during pregnancy was associated with reduced risks of stillbirth or newborn death, SGA. The protective effect may be due to its effect against SARS-CoV-2 infection. COVID-19 vaccination during pregnancy was not associated with any of the adverse neonatal or maternal outcomes of interest in this study. However, the conclusions are mainly limited by the types and timing of vaccination. The vaccinations in our study received during pregnancy were primarily mRNA vaccines administered in the second and third trimester. The studies included for data synthesis in this study areobservational. Future RCTs are warranted to further assess the safety and efficacy of COVID-19 vaccination in pregnancy and to evaluate long-term effects. The systematic review and meta-analysis will be updated when relevant new evidence becomes available.

## Data availability statement

The original contributions presented in the study are included in the article/Supplementary material, further inquiries can be directed to the corresponding author.

## Author contributions

CD and YL conceptualized and designed the study, collected data, and drafted the initial manuscript. WP, DZ, and KW participated in analysis and interpretation of results and revised the manuscript. YC was the senior investigators and conceptualized and contributed to data collection, carried out the statistical analysis, and reviewed and revised the manuscript. All authors approved the final manuscript as submitted and agree to be accountable for all aspects of the work.
