# Supplementary material for: Associations of COVID-19 vaccination during pregnancy with adverse neonatal and maternal outcomes: A systematic review and meta-analysis
Source: Front Public Health. 2023 Jan 30;11:1044031. doi: 10.3389/fpubh.2023.1044031 (PMC9922836; doi:10.3389/fpubh.2023.1044031)
Supplement: Supplementary file 1 [file Data_Sheet_1.PDF]

Supplementary material

Methods S1. Search Strategy.

Table S1. Characteristics of the included studies for data synthesis.

Table S2. Characteristics of the included studies for qualitative synthesis (not for data synthesis).

Table S3. Quality assessment of included cohort studies via Newcastle-Ottawa Scale.

Table S4. Quality assessment of included case-control studies via Newcastle-Ottawa Scale.

Table S5. Sensitivity analysis restricted to studies comprising participants without COVID-19 after vaccination.

Table S6. Sensitivity analysis restricted to studies controlled potential confounders.

Table S7. Sensitivity analysis restricted to studies with all pregnant people vaccinated in the first trimester.

Table S8. Sensitivity analysis restricted to studies of high quality.

Table S9. Subgroup analysis of association between COVID-19 vaccination during pregnancy and NICU admission or hospitalization.

Table S10. Subgroup analysis of association between COVID-19 vaccination during pregnancy and birthweight.

Table S11. Subgroup analysis of association between COVID-19 vaccination during pregnancy and gestational age at delivery.

Table S12. Subgroup analysis of association between COVID-19 vaccination during pregnancy and cesarean delivery.

Figure S1. Forest plot for congenital anomalies.

Figure S2. Forest plot for small for gestational age.

Figure S3. Forest plot for small for NICU admission or hospitalization.

Figure S4. Forest plot for Apgar score < 7 at 5 minutes.

Figure S5. Forest plot for low birth weight.

Figure S6. Forest plot for birth weight (g).

Figure S7. Forest plot for gestational age at delivery (weeks).

Figure S8. Forest plot for miscarriage.

Figure S9. Forest plot for cesarean delivery.

Figure S10. Forest plot for postpartum hemorrhage.

Figure S11. Forest plot for chorioamnionitis.

Figure S12. Forest plot for placental abruption.

Figure S13. Forest plot for maternal ICU admission.

Method S1. Search Strategy.

Embase

Embase

No.	Query Results	Results	Date
#1.	pregnan*:ab,kw,ti	759620	25 May 2022
#2.	prenatal:ab,kw,ti	141889	
#3.	maternal:ab,kw,ti	377525	
#4.	antenatal:ab,kw,ti	58874	
#5.	gestation*:ab,kw,ti	324680	
#6.	#1 OR #2 OR #3 OR #4 OR #5	1130733	
#7.	vaccin*:ab,kw,ti	449640	
#8.	immuniz*:ab,kw,ti	170059	
#9.	#7 OR #8	537963	
#10.	coronavirus:ab,kw,ti	101263	
#11.	'covid 19':ab,kw,ti	239308	
#12.	'sars-cov-2':ab,kw,ti	89941	
#13.	#10 OR #11 OR #12	276366	
#14.	#6 AND #9 AND #13	971	
#15.	#6 AND #9 AND #13 AND [2020-2022]/py	922	
#16.	#6 AND #9 AND #13 AND [2020-2022]/py AND [review]/lim	156	
#17.	#15 NOT #16	766	
#18.	#15 NOT #16 AND ([animal cell]/lim OR [animal experiment]/lim OR [animal model]/lim OR [animal		

tissue]/lim) AND [animals]/lim 14

#19. #17 NOT #18 752

MEDLINE via Ovid

Ovid MEDLINE(R) ALL <1946 to May 25, 2022>

1 pregnan*.mp. 1085843

2 "pregnan*".m_titl. 251383

3 prenatal.mp. 191385

4 "prenatal".m_titl. 43326

5 maternal.mp. 347943

6 "maternal".m_titl. 89193

7 "antenatal".m_titl. 11287

8 antenatal.mp. 41588

9 gestation*.mp. 272126

10 "gestation*".m_titl. 38731

11 1 or 2 or 3 or 4 or 5 or 6 or 7 or 8 or 9 or 10 1289961

12 vaccin*.mp. 440010

13 "vaccin**".m_titl. 197117

14 immuniz*.mp. 197756

15 "immuniz*".m_titl. 35777

16 12 or 13 or 14 or 15 536084

17 coronavirus.mp. 129804

18 "coronavirus".m_titl. 25298

19 covid-19.mp. 248695

20 "covid-19".m_titl. 169278

21 sars-cov-2.mp. 162851

22	"sars-cov-2".m_titl.	39046
23	17 or 18 or 19 or 20 or 21 or 22	272058
24	11 and 16 and 23	1026
25	limit 24 to "review articles"	183
26	24 not 25	843
27	limit 26 to yr="2020 -Current"	754
28	limit 27 to animals	22
29	27 not 28	732

Cochrane Database of Systematic Reviews (CDSR)

Date Run: 25/05/2022 14:49:20

Comment:

ID	Search	Hits
#1	pregnan*	75562
#2	maternal	26825
#3	prenatal	7901
#4	antenatal*	5666
#5	gestation*	29015
#6	#1 or #2 or #3 or #4 or #5	94221
#7	coronavirus	6231
#8	covid-19	10385
#9	sars-cov-2	441
#10	#7 or #8 or #9	10953
#11	immuniz*	9147
#12	vaccin*	29091
#13	#11 or #12	30802

#14 #6 and #10 and #13 179

Table S1. Characteristics of the included studies for data synthesis.

Source	Country	Vaccine type, number	Timing of first dose vaccine, number (%)	Outcomes of interest	Control
Beharier et al,[16] 2021	Israel.	92 (100%) BNT162b2.	Not Specified	Birthweight, NICU admission, preterm delivery, gestational age at delivery.	Unvaccinated pregnancies without prior documentation for infection.
Blakeway et al,[17] 2022	United Kingdom	109 (77.8%) BNT162b2; 18 (12.9%) mRNA-1273; 13 (9.3%) AZD1222.	20 (14.3%) in the second trimester. 120 (85.7%) in the third trimester;	Neonatal birthweight, small for gestational age, fetal abnormalities, stillbirth, NICU admission; postpartum hemorrhage, cesarean delivery, maternal high-dependency unit admission.	Did not receive a vaccine during pregnancy.
Bleicher et al,[18] 2021	Israel (Online questionnaire)	COVID-19 vaccine (not specified).	36 (17.8%) in the first trimester; 110 (54.5%) in the second trimester; 56 (27.7%) in the third trimester.	Preterm birth, anomaly on anatomy scan, pregnancy loss up to 13 gestational week.	Unvaccinated pregnant women
Citu(1) et al,[19] 2022	Romania	638 (68.8%) BNT162b2; 289 (31.2%) mRNA-1273	The first trimester.	Spontaneous abortion	Not vaccinate against SARS-CoV-2 during first trimester of pregnancy.
Citu(2) et al,[20] 2022	Romania	115 (66.4%) BNT162b2; 58 (33.6%) Ad26.COV2.S	The third trimester.	Small for gestational age, Apgar score < 7 at 5 min, preterm delivery, cesarean delivery, placental abruption, postpartum hemorrhage.	Unvaccinated seronegative pregnant people without COVID-19.

Conti et al,[35] 2022	Italy	11 (100%) BNT162b2	5 (45%) in the second trimester; 6 (55%) in the third trimester.	Birthweight, gestational age at delivery.	Lactating women who received vaccine after delivery.
Dick et al,[21] 2022	Israel	BNT162b2 or mRNA-1273 (not specified).	12 (0.5%) in the first trimester; 964 (41.8%) in the second trimester; 1,329 (57.7%) in the third trimester.	Preterm birth, small for gestational age, gestational age at delivery, intrauterine fetal demise, birthweight, Apgar score < 7 at 5 min, cesarean delivery, postpartum hemorrhage.	Unvaccinated singleton pregnant people without COVID-19 infection.
Fell et al,[22] 2022	Canada	18101 (79.9%) BNT162b2; 4507 (19.9%) mRNA-1273; 52 (0.2%) others.	201 (0.9%) in the first trimester; 8047 (35.5%) in the second trimester; 14412 (63.6%) in the third trimester.	NICU admission, Apgar score < 7 at 5 min, Postpartum hemorrhage, Chorioamnionitis, Cesarean delivery	Not vaccinated at any point.
Goldshtein et al,[23] 2021	Israel	7530 (100%) BNT162b2.	Not specified.	Stillbirth, preterm birth, birthweight, birth week, maternal death, abortion.	Unvaccinated on the day of matching and had no prior COVID-19.
Goldshtein et al,[24] 2022	Israel	16697 (100%) BNT162b2.	2134 (12.8%) in the first trimester; 9364 (56.1%) in the second trimester; 5199 (31.1%) in the third trimester.	SGA, LBW, neonatal hospitalization, congenital anomalies, preterm birth, gestational age at delivery, birth weight.	Unvaccinated people with singleton pregnancy.
Kazan et al,[25] 2022	USA	COVID-19 vaccine (not specified).	Not specified.	Preterm birth.	No history of COVID disease or vaccine.

Kharbanda et al,[26] 2021	USA	7.8% of 105446 BNT162b2; 6% of 105446 mRNA-1273; 0.5 % of 105446 Ad26.COV.2.S.	Before 20 weeks' gestation.	Spontaneous abortion.	No vaccination within 28 days at presentation.
Lipkind et al,[27] 2022	USA	5,478 (54.4%) BNT162b2; 4,162 (41.4%) mRNA-1273; 424 (4.2%) Ad26.COV.2.S.	172 (1.7%) in the first trimester; 3,668 (36.5%) in the second trimester; 6,224 (61.8%) in the third trimester.	Preterm birth, SGA.	Not vaccinated with COVID-19 from the last menstrual period through 3 days before delivery.
Magnus et al,[28] 2021	Norway	790 (78.8%) BNT162b2; 137 (13.7%) mRNA-1273; 76 (7.6%) AZD1222.	The first trimester.	Miscarriage.	Unvaccinated pregnant women.
Magnus et al,[29] 2022	Sweden, Norway	20424 (71.6%) BNT162b2; 7607 (26.7%) mRNA-1273; 475 (1.7%) AZD1222.	1125 (3.9%) in the first trimester; 13012 (45.6%) in the second trimester; 14368 (50.4%) in the third trimester.	Stillbirth, AGA, preterm birth, Apgar score < 7 at 5 min, NICU admission.	Singleton pregnancy, not vaccinated before the date of delivery.
Matsui et	USA	17 (56.7%)	5 (16.7%) in the first trimester;	Preterm, gestational age at delivery.	Unvaccinated with no evidence of

al,[36] 2022		BNT162b2; 13 (43.3%) mRNA-1273.	12 (40.0%) in the second trimester; 13 (43.3%) in the third trimester.		prior infection.
Peretz-Machluf et al,[37] 2022	Israel	3240 (100%) BNT162b2	Not specified.	SGA, preterm birth, gestational age at delivery, cesarean delivery, birthweight, Apgar score < 7 at 5 min, NICU admission.	Non-vaccinated pregnant women with singleton pregnancies.
Rottenstreich et al,[30] 2022	Israel	712 (100%) BNT162b2	The third trimester.	Intrauterine fetal death, preterm birth, SGA, Apgar score < 7 at 5 min, NICU admission, birthweight, gestational age at delivery, placental abruption, postpartum haemorrhage, chorioamnionitis, maternal ICU admission, caesarean delivery.	Unvaccinated pregnant people gave birth at >24 weeks of gestation,without Covid-19 history.
Ruderman et al,[31] 2022	USA	mRNA vaccine and viral vector vaccine (not specified).	The first trimester.	Congenital fetal anomalies identified on Ultrasonography.	Unvaccinated pregnant people.
Shanes et al,[32] 2021	USA	COVID-19 vaccine (not specified).	Not specified.	Gestational age at delivery.	Unvaccinated pregnant people without COVID-19.
Stock et al,[12] 2002	United Kingdom	20,572 (79.4%) BNT162b2; 3,224 (12.4%) mRNA-1273; 2,121 (8.2%) AZD1222.	9,905 (38.2%) in the first trimester; 9,317 (35.9%) in the second trimester; 6,695 (25.8%) in the third trimester.	Stillbirth or neonatal death, preterm birth.	Unvaccinated people and gave birth.
Theiler et al,[33] 2021	USA	127 (90.7%) BNT162b2; 12	From 13.9 to 40.6 weeks' gestation.	Stillbirth, neonatal ICU admission, Apgar score < 7 at 5 min, preterm birth, LBW, postpartum hemorrhage,	No COVID-19 vaccination during pregnancy

		(8.6%) mRNA-1273; 1 (0.7%) Ad26.COV.2.S.		cesarean delivery, maternal ICU admission.	
Wainstock et al,[34] 2021	Israel	913 (100%) BNT162b2.	The second or third trimester.	SGA, birthweight, Apgar < 7 at 5 min, gestational age at delivery, placental abruption, postpartum hemorrhage, cesarean delivery.	Singleton pregnancy without COVID-19.

Table S2. Characteristics of the included studies for qualitative synthesis (not for data synthesis).

Source	Country	Study design	Type of vaccine	Timing of first dose vaccine	Outcomes of interest	Control	Without SARS-Cov-2 infection	Separated results for the first trimester
Ben-Mayor 2022	Israel	Cohort	58 (100%) BNT162b2	58 (100%) 3rd trimester	Gestational age at delivery, cesarean delivery, birth weight<br>Apgar 5 min <7, admission to NICU.	-	Yes	No.
Bookstein Peretz 2021	Israel	case–control	390 (100%) BNT162b2	76 (19.5%) 1st trimester<br>193 (49.5%) 2nd trimester<br>121 (31.0%) 3rd trimester	Gestational age at birth, birth weight, SGA, placental abruption, placental abruption, postpartum hemorrhage, NICU hospitalization.	Non-pregnant people	Not specified.	No.
Gray 2021	USA	Cohort	47 (50%) BNT162b2; 47 (50%) mRNA-1273.	11 (13%) 1st trimester<br>39 (46%) 2nd trimester<br>34 (40%) 3rd trimester	Gestational age at delivery, cesarean, birthweight, preterm delivery, NICU admission.	Nonpregnant; Lactating; Pregnant with previous COVID-19.	Not specified.	No.
Kachikis 2021	US	Cohort	4777 (61.5%) BNT162b2; 2970 (38.2%) mRNA-1273;<br>23 (0.3%) JNJ-78436735.	1822 (23.9%) 1st trimester<br>3694 (48.5%) 2nd trimester<br>2095 (27.5%) 3rd trimester	Miscarriages.	Lactating individuals<br>Individuals planning pregnancy	Not specified.	No.

Kashani-Ligumsky 2021	Israel	Cohort	29 (100%) BNT162b2.	The third trimester.	Gestational age at delivery, preterm delivery, birth weight.	Unvaccinated, with out COVID-19 during pregnancy.	Yes	NO.
Kugelman 2022	Israel	Cohort	130 (100%) BNT162b2.	The second trimester	Gestational age at birth, newborn weight.	-	Yes	No
Moro 2022	USA	Cohort	1831 (52.9%) BNT162b2; 1350 (39.0%) mRNA-1273; 275 (7.9%) Ad26.COV2.S.	1,040 (45.5%) 1st trimester 727 (31.8%) 2nd trimester 520 (22.7%) 3rd trimester	Preterm delivery, stillbirth, maternal deaths, neonatal death, birth defects, infant in intensive care unit, spontaneous abortion.	-	No	No
Nir 2022	Israel	Cohort	64 (100%) BNT162b2.	Not specified	Gestational age at delivery, neonatal birthweight.	Pregnant people recoverd from COVID-19.	Not specified	No
Rottenstreich(2) 2022	Israel	Cohort	422 (100%) BNT162b2.	90 (22.4%) 1st trimester 124 (30.8%) 2nd trimester 188 (46.8%) 3rd trimester	Preterm, cesarean delivery, neonatal birthweight, gestational age at delivery.	-	Yes	Yes
Santos 2022	USA	Cohort	31 (64.6%) BNT162b2; 17 (35.4%) mRNA-1273.	Not specified	Preterm, miscarriage, SGA, gestational age at delivery, post partum hemorrhage.	-	Yes	No
Shen 2022	China	Cohort	29 (100%) mRNA-1273.	1 (3.4%) 2nd trimester 28 (96.6%) 3rd trimester	gestation age at delivery, weight of newborn	-	Yes	No

Shimabukuro 2021	USA	Cohort	2136 (54.0%) BNT162b2; 1822 (46.0%) mRNA-1273.	1132 (28.6%) 1st trimester; 1714 (43.3%) 2nd trimester; 1019 (25.7%) 3rd trimester.	Gestational age at delivery, cesarean, birthweight, preterm delivery, NICU admission, miscarriages.	Published incidence.	No	No
Shook 2022	USA	Cohort	52 (68%) BNT162b2; 25 (32%) mRNA-1273.	20 to 32 weeks' gestation	Gestational age at delivery, birth weight.	Symptomatic SARS-CoV-2 infection in pregnancy.	Yes	No
Sourouni 2022	Germany	Cohort	62 (89%) BNT162b2; 7 (10%) mRNA-1273; 1 (1%) unknown.	7 (10%) 1st trimester; 21 (30%) 2nd trimester; 42 (60%) 3rd trimester.	Fetal malformation.	-	Not specified	No
Sukhikh 2021	Rusia	Cohort	708 (91.6%) Gam-COVID-Vac; 7 (0.9%) CoviVac; 27 (3.5%) EpiVacCorona; 31 (4.0%) Sputnik Light.	683 (88.4%) < 12 weeks' gestation; 51(6.6%) 12 to 22 weeks' gestation; 39 (5%) > 22 weeks' gestation.	Fetal malformations	-	No	No
Trostle 2021	USA	Cohort	332 (78.3%) BNT162b2; 92 (21.7%) mRNA-1273.	124 (29.2%) 1st trimester; 193 (45.5%) 2nd trimester; 107 (25.2%) 3rd trimester.	Preterm delivery, cesarean delivery, abruption, NICU admission, birthweight SGA, congenital anomalies, gestational age at delivery.	-	Yes	No

Vuong 2022	Viet Nam	Cohort	441 (46.2%) AstraZeneca<br>513 (53.8%) BNT162b2	Approximately 30 to 31 weeks' gestation	Preterm delivery, stillbirth, maternal death, birth weight, low birth weight, birthweight, NICU admission, gestational age at birth.	-	No	No
Yang 2021	USA	Cohort	992 (75.1%) BNT162b2;<br>296 (22.4%) mRNA-1273;<br>33 (2.5%) JNJ-78436735	193 (14.6%) 1st trimester;<br>699 (52.9%) 2nd trimester;<br>429 (32.5%) 3rd trimester.	Gestational age at delivery.	-	No	Yes
Zauche 2021	USA	Cohort	1294 (52.7%) BNT162b2;<br>1162 (47.3%) mRNA-1273;	1230 (50.1%) 1st trimester;<br>846 (34.4%) 2nd trimester.	Spontaneous abortion.	-	Not specified.	Yes
Zdanowski 2021	Poland	Cohort	16 (100%) BNT162b2	The 3rd trimester	Birth weight, gestational age at delivery.	-	Yes	No

Table S3. Quality assessment of included cohort studies via Newcastle-Ottawa Scale.

**Source**	**Selection**				**Comparability**	Outcome			Total
	Representativeness of the exposed cohort	Selection of the non-exposed cohort	Ascertainment of exposure	Demonstration that outcome of interest was not present at the start of the study	Comparability of cohorts on the basis of the design or analysis	Assessment of outcome	Was follow-up long enough for outcomes to occur	Adequacy of the follow-up of cohorts	
Beharier 2021	1	1	1	1	A (1) B (1)	1	1	1	9
Blakeway 2022	1	1	1	1	A (1) B (1)	1	1	1	9
Bleicher 2021	0	1	0	1	A (0) B (0)	0	0	0	2
Citu(1) 2022	1	1	1	1	A (0) B (0)	1	1	1	7
Citu(2) 2022	1	1	1	1	A (0) B (1)	1	1	1	8
Conti 2022	1	0	1	0	A (0) B (0)	1	1	1	5
Dick 2022	1	1	1	1	A (1) B (0)	1	1	1	8
Fell 2022	1	1	1	1	A (1) B (1)	1	1	1	9

Goldshtein 2021	1	1	1	1	A (1)<br>B (0)	1	0	1	7
Goldshtein 2022	1	1	1	1	A (1)<br>B (1)	1	1	1	9
Kazan 2022	1	1	1	1	A (0)<br>B (0)	0	1	0	5
Lipkind 2022	1	1	1	1	A (1)<br>B (0)	1	1	1	8
Magnus 2022	1	1	1	1	A (1)<br>B (1)	1	1	1	9
Matsui 2022	1	0	1	0	A (0)<br>B (1)	1	1	1	6
Peretz-Machluf 2022	1	1	1	1	A (0)<br>B (1)	1	1	1	8
Rottenstreich 2022	1	1	1	1	A (1)<br>B (1)	1	1	1	9
Ruderman 2022	1	1	1	0	A (1)<br>B (0)	1	1	1	7
Shanes 2021	0	1	1	1	A (0)<br>B (0)	1	1	1	6
Stock 2002	1	1	1	1	A (0)<br>B (1)	1	1	1	8
Theiler 2021	1	1	1	1	A (0)<br>B (1)	1	1	1	8

Wainstock 2021	1	1	1	1	A (1)<br>B (0)	1	1	1	8

For comparability: A is age, B is SARS-CoV-2 infection.

Table S4. Quality assessment of included case-control studies via Newcastle-Ottawa Scale.

Source	Selection				Comparability	Outcome			Total
	Is the case definition adequate	Representativeness of the cases	Selection of Controls	Definition of Controls	Comparability of cases and controls on the basis of the design or analysis	Ascertainment of exposure	Same method of ascertainment for cases and controls	Non-Response rate	
Kharbanda 2021	0	1	1	1	A(1)<br>B(0)	1	1	1	7
Magnus 2021	0	1	1	1	A(1)<br>B(1)	1	1	1	8

For comparability: A is age, B is SARS-CoV-2 infection.

Table S5. Sensitivity analysis restricted to studies comprising participants without COVID-19 after vaccination.

	Studies	Participants	OR	95%CI	*P* value	I^2
Stillbirth or neonatal death	4	9707	0.95	0.58 - 1.56	0.85	0
Congenital anomalies	1	524	0.81	0.22 - 2.96	0.75	-
Preterm birth	6	30783	0.88	0.78 - 0.99	0.03	0
Small for gestational age	6	33882	0.97	0.85 - 1.09	0.57	15
Low birth weight	2	22654	0.89	0.77 - 1.03	0.12	0
APGAR(5min)<7	5	14284	0.97	0.72 - 1.31	0.84	0
NICU admission or hospitalization	4	24953	1.00	0.88 - 1.14	0.97	75
Birthweight	5	12517	MD, -14.54 g	MD, -53.84 - 24.76 g	0.47	70
Gestational age at delivery	6	12049	MD, -0.13 w	MD, -0.29 - 0.04 w	0.13	73
Cesarean delivery	6	14808	1.09	0.92 - 1.28	0.33	55
Postpartum hemorrhage	6	14808	0.98	0.80 - 1.20	0.84	2
Chorioamnionitis	1	1775	0.80	0.41 - 1.54	0.51	-
Maternal ICU admission	2	2314	2.36	0.96 - 5.78	0.06	0
Placental abruption	3	6876	0.66	0.38 - 1.16	0.15	0

Table S6. Sensitivity analysis restricted to studies controlled potential confounders.

	Studies	Participants	OR	95%CI	*P* value	I^2
Stillbirth or neonatal death （OR)	3	21210	0.77	0.42 - 1.38	0.37	0
Stillbirth or neonatal death （HR)	1	157521	0.86	0.63 - 1.17	-	-
Congenital anomalies	2	6250	0.83	0.58 - 1.19	0.31	0
Preterm birth (OR)	4	32780	0.88	0.79 - 0.98	0.02	0
Preterm birth (HR)	1	157521	0.98	0.91 - 1.05	-	-
Small for gestational age	6	194467	0.96	0.90 - 1.01	0.14	0
Low birth weight	1	22697	0.87	0.76 - 0.99	0.03	-
APGAR(5min)<7	3	215061	0.93	0.86 - 1.01	0.10	0
NICU admission or hospitalization	6	238956	0.93	0.89 - 0.98	0.003	0
Birthweight	2	2972	MD, 3.05 g	MD, -31.38-37.47 g	0.86	0
Gestational age at delivery	2	2972	MD, 0.001 w	MD, -0.05-0.053 w	0.96	0
Miscarriage	3	138983	0.96	0.81 - 1.13	0.62	73
Cesarean delivery	4	63324	0.96	0.93 - 0.99	0.006	0
Postpartum hemorrhage	4	63324	0.99	0.83 - 1.18	0.90	22
Chorioamnionitis	1	52775	1.20	0.90 - 1.59	-	-
Maternal ICU admission	1	532	1.53	0.64 - 3.67	0.34	-

Table S7. Sensitivity analysis restricted to studies with all pregnant people vaccinated in the first trimester.

	Studies	Participants	OR	95%CI	*P* value	I^2
Congenital anomalies	2	6976	0.73	0.52 - 1.01	0.06	0
Preterm birth	2	135742	1.07	0.81 - 1.41	0.64	62
Small for gestational age	2	135742	1.01	0.87 - 1.17	0.93	0
Low birth weight	1	5602	1.07	0.85 - 1.35	0.56	-
APGAR(5min)<7	1	130140	1.23	0.80 - 1.88	0.34	-
NICU admission or hospitalization	2	135742	1.05	0.72 - 1.54	0.80	83
Miscarriage	2	21571	1.03	0.89 - 1.20	0.65	0

Table S8. Sensitivity analysis restricted to studies of high quality.

	Studies	Participants	OR	95%CI	*P* value	I^2
Stillbirth or neonatal death	7	264974	0.74	0.60 - 0.92	0.007	0
Congenital anomalies	3	9097	0.82	0.62- 1.08	0.16	0
Preterm birth	9	279206	0.98	0.90 - 1.06	0.60	36
Small for gestational age	8	197083	0.95	0.86 - 1.04	0.24	39
NICU admission or hospitalization	8	242831	0.94	0.84 - 1.04	0.22	70
APGAR(5min)<7	8	227639	0.93	0.86 - 1.01	0.07	0
Low birth weight	2	24838	1.00	0.88 - 1.14	0.98	0
Birthweight	8	84187	MD, 3.82 g	MD, -9.49 - 17.13 g	0.57	46
Gestational age at delivery	7	71239	MD, -0.05 w	MD, -0.10 - 0.01 w	0.11	67
Miscarriage	3	36631	0.99	0.88 - 1.11	0.84	0
Postpartum hemorrhage	7	67,803	0.91	0.81 - 1.01	0.08	5
Cesarean delivery	7	67803	1.10	1.00 - 1.22	0.06	47
Chorioamnionitis	2	54550	1.18	0.65 - 2.13	0.59	66
Maternal ICU admission	2	2534	2.05	0.65 - 6.50	0.22	22
Placental abruption	3	6876	0.66	0.38 - 1.16	0.15	0

Table S9. Subgroup analysis of association between COVID-19 vaccination during pregnancy and NICU admission or hospitalization.

	Subgroup	Studies	Participants	OR	95%CI	*P* value	I^2
Overall		8	239131	0.94	0.84 - 1.05	0.28	75
WHO geographic region	America	3	58557	0.84	0.80 - 0.89	<0.001	0
	Europe	5	184274	0.99	0.95 - 1.04	0.75	0
Source of outcome data	Medical records	4	6165	0.90	0.65 - 1.24	0.51	0
	Registry	4	23666	0.94	0.83 - 1.06	0.31	86

Table S10. Subgroup analysis of association between COVID-19 vaccination during pregnancy and birthweight.

	Subgroup	Studies	Participants	Mean difference, g	95%CI	*P* value	I^2
Overall		9	84,210	0.81	-15.55 - 17.18	0.92	59
WHO geographic region	America	1	52775	17	7.69 - 26.31	< 0.001	-
	Europe	8	31435	- 5.78	-26.63 - 15.06	0.59	51
Source of outcome data	Medical records	7	16375	9.44	-7.12 - 26.00	0.26	73
	Registry	2	67835	-11.24	-42.26 - 19.78	0.48	58

Table S11. Subgroup analysis of association between COVID-19 vaccination during pregnancy and gestational age at delivery.

	Subgroup	Studies	Participants	Mean difference, week	95%CI	*P* value	I^2
Overall		10	71,496	-0.05	-0.11 - 0.01	0.10	63
WHO geographic region	America	3	53009	0.00	-0.03-0.03	0.99	0
	Europe	7	18487	-0.08	-0.18-0.01	0.09	73
Source of outcome data	Medical records	8	15907	-0.10	-0.23 - 0.02	0.11	63
	Registry	2	55589	0.00	-0.03 - 0.03	1.00	0

Table S12. Subgroup analysis of association between COVID-19 vaccination during pregnancy and cesarean delivery.

	Subgroup	Studies	Participants	Odds ratio	95%CI	*P* value	I^2
Overall		8	71,947	1.07	0.96 - 1.19	0.25	59
WHO geographic region	America	2	54777	1.12	1.08 - 1.16	< 0.001	0
	Europe	6	17170	1.04	0.88 - 1.24	0.64	66
Source of outcome data	Medical records	6	17170	1.04	0.88 - 1.24	0.64	66
	Registry	2	54777	1.12	1.08 - 1.16	< 0.001	0

Study or Subgroup	Vaccinated Events	Vaccinated Total	Unvaccinated Events	Unvaccinated Total	Weight	Odds Ratio IV, Random, 95% CI
Blakeway et al,[17] 2022	3	133	10	399	4.2%	0.90 [0.24, 3.31]
Bleicher et al,[18] 2021	9	202	6	124	6.4%	0.92 [0.32, 2.64]
Goldshtein et al,[24] 2022	43	2134	87	3584	52.3%	0.83 [0.57, 1.20]
Ruderman et al,[31] 2022	95	2313	27	534	37.2%	0.80 [0.52, 1.25]
**Total (95% CI)**		**4782**		**4641**	**100.0%**	**0.83 [0.63, 1.08]**
Total events	150		130			

Heterogeneity: $Tau^2 = 0.00$; $Chi^2 = 0.07$, df = 3 (P = 1.00); $I^2 = 0\%$

Test for overall effect: Z = 1.40 (P = 0.16)

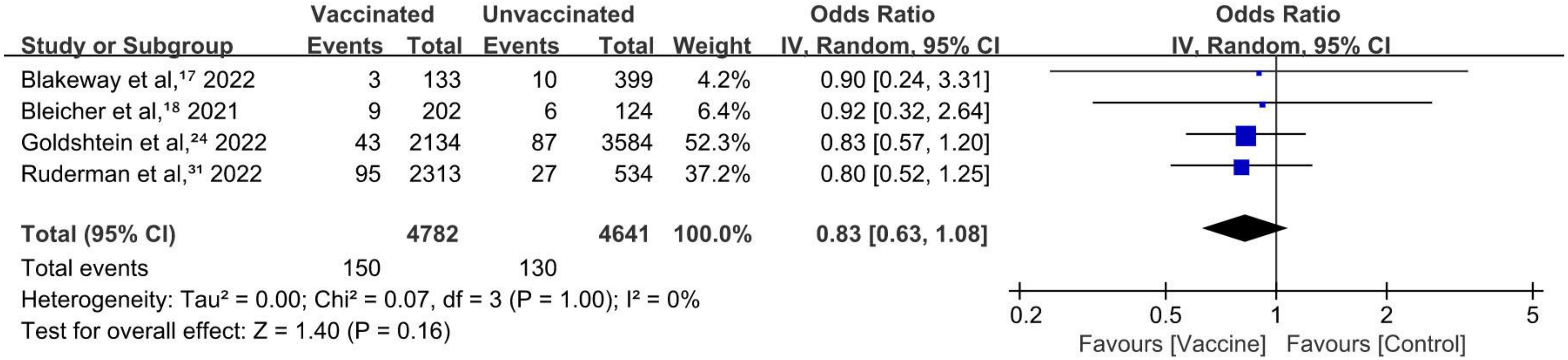

Figure S1. Forest plot for congenital anomalies.

Study or Subgroup	Vaccinated Events	Vaccinated Total	Unvaccinated Events	Unvaccinated Total	Weight	Odds Ratio IV, Random, 95% CI
Blakeway et al,[17] 2022	16	133	48	399	2.3%	1.00 [0.55, 1.83]
Citu(2) et al,[20] 2022	6	173	26	529	1.0%	0.70 [0.28, 1.72]
Dick et al,[21] 2022	142	2305	233	3313	13.0%	0.87 [0.70, 1.08]
Goldshtein et al,[24] 2022	1030	15864	468	6972	26.3%	0.96 [0.86, 1.08]
Magnus et al,[29] 2022	2143	28506	10821	129015	38.3%	0.89 [0.85, 0.93]
Peretz-Machluf et al,[37] 2022	417	3240	49	460	7.3%	1.24 [0.91, 1.70]
Rottenstreich et al,[30] 2022	81	712	98	1063	7.4%	1.26 [0.93, 1.73]
Wainstock et al,[34] 2021	26	913	131	3486	4.3%	0.75 [0.49, 1.15]
**Total (95% CI)**		**51846**		**145237**	**100.0%**	**0.95 [0.86, 1.04]**
Total events	3861		11874			

Heterogeneity: Tau² = 0.01; Chi² = 11.43, df = 7 (P = 0.12); I² = 39%
Test for overall effect: Z = 1.18 (P = 0.24)

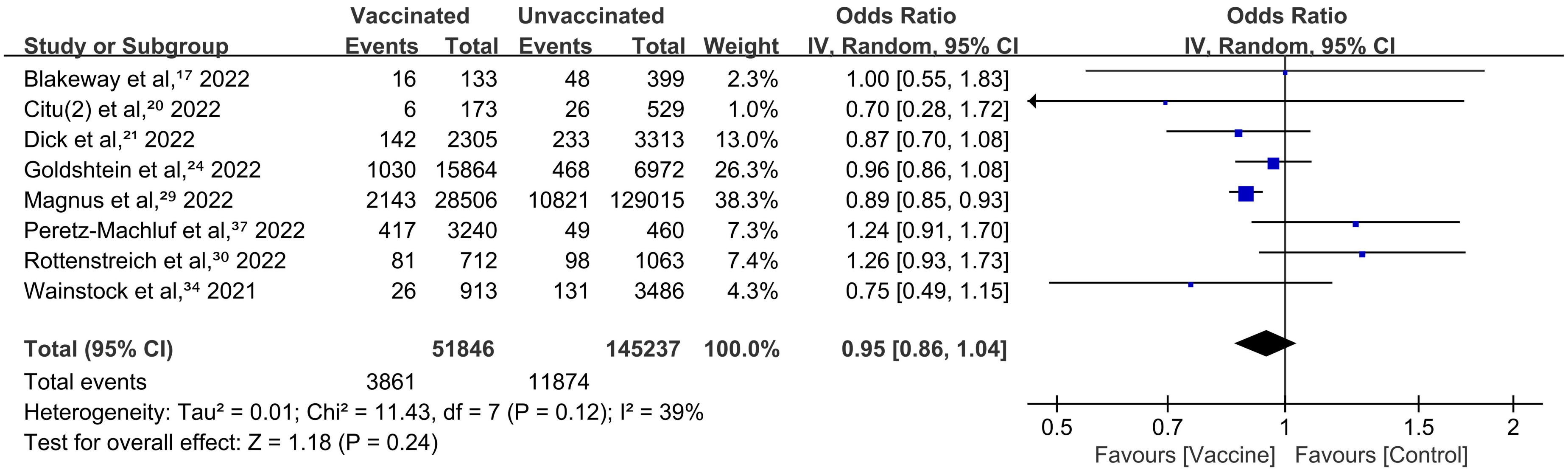

Figure S2. Forest plot for small for gestational age.

	Vaccinated		Unvaccinated			Odds Ratio
Study or Subgroup	Events	Total	Events	Total	Weight	IV, Random, 95% CI
Beharier et al,[16] 2021	4	92	1	66	0.2%	2.95 [0.32, 27.06]
Blakeway et al,[17] 2022	7	133	20	399	1.4%	1.05 [0.43, 2.55]
Fell et al,[22] 2022	2508	22746	3852	30109	32.6%	0.84 [0.80, 0.89]
Goldshtein et al,[24] 2022	916	16697	416	7591	24.5%	1.00 [0.89, 1.13]
Magnus et al,[29] 2022	2418	28506	11021	129015	33.3%	0.99 [0.95, 1.04]
Peretz-Machluf et al,[37] 2022	89	3240	16	460	3.4%	0.78 [0.46, 1.35]
Rottenstreich et al,[30] 2022	29	712	48	1063	4.4%	0.90 [0.56, 1.44]
Theiler et al,[33] 2021	1	140	11	1862	0.3%	1.21 [0.16, 9.44]
**Total (95% CI)**		**72266**		**170565**	**100.0%**	**0.94 [0.84, 1.04]**
Total events	5972		15385			

Heterogeneity: Tau² = 0.01; Chi² = 23.08, df = 7 (P = 0.002); I² = 70%

Test for overall effect: Z = 1.24 (P = 0.22)

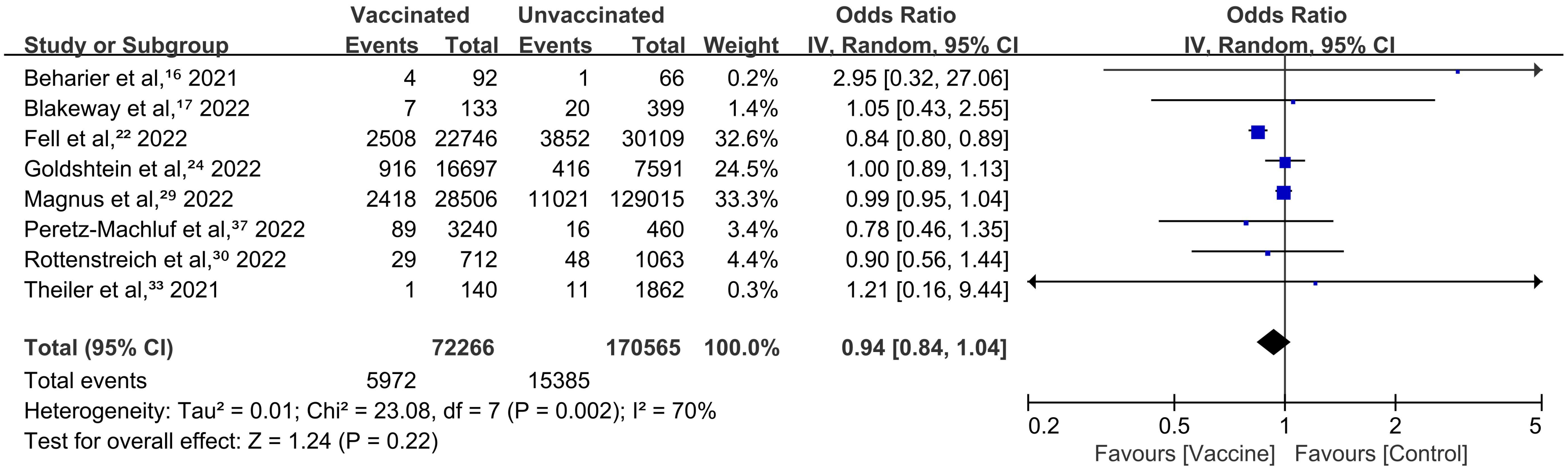

Figure S3. Forest plot for NICU admission or hospitalization.

Study or Subgroup	Vaccinated Events	Vaccinated Total	Unvaccinated Events	Unvaccinated Total	Weight	Odds Ratio IV, Random, 95% CI
Citu(2) et al,[20] 2022	2	173	5	529	0.2%	1.23 [0.24, 6.38]
Dick et al,[21] 2022	42	2305	63	3313	3.9%	0.96 [0.65, 1.42]
Fell et al,[22] 2022	403	22334	588	29588	37.4%	0.91 [0.80, 1.03]
Magnus et al,[29] 2022	429	28506	2060	129015	55.7%	0.94 [0.85, 1.05]
Peretz-Machluf et al,[37] 2022	10	3240	1	460	0.1%	1.42 [0.18, 11.13]
Rottenstreich et al,[30] 2022	21	712	27	1063	1.8%	1.17 [0.65, 2.08]
Theiler et al,[33] 2021	3	140	38	1862	0.4%	1.05 [0.32, 3.45]
Wainstock et al,[34] 2021	2	913	30	3486	0.3%	0.25 [0.06, 1.06]
**Total (95% CI)**		**58323**		**169316**	**100.0%**	**0.93 [0.86, 1.01]**
Total events	912		2812			

Heterogeneity: Tau² = 0.00; Chi² = 4.30, df = 7 (P = 0.74); I² = 0%

Test for overall effect: Z = 1.81 (P = 0.07)

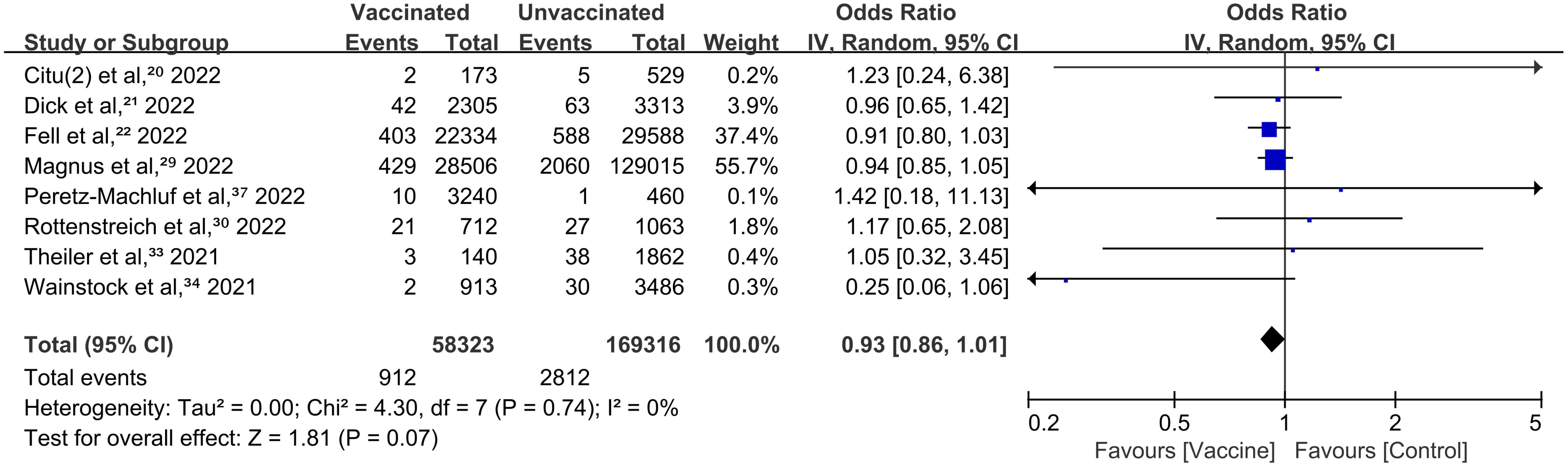

Figure S4. Forest plot for Apgar score < 7 at 5 minutes.

Study or Subgroup	Vaccinated Events	Vaccinated Total	Unvaccinated Events	Unvaccinated Total	Weight	Odds Ratio IV, Random, 95% CI
Goldshtein et al,[24] 2022	730	15864	324	6972	95.8%	0.99 [0.87, 1.13]
Theiler et al,[33] 2021	11	140	121	1862	4.2%	1.23 [0.65, 2.33]
**Total (95% CI)**		**16004**		**8834**	**100.0%**	**1.00 [0.88, 1.14]**
Total events	741		445			

Heterogeneity: $Tau^2 = 0.00$; $Chi^2 = 0.41$, df = 1 (P = 0.52); $I^2 = 0\%$

Test for overall effect: Z = 0.02 (P = 0.98)

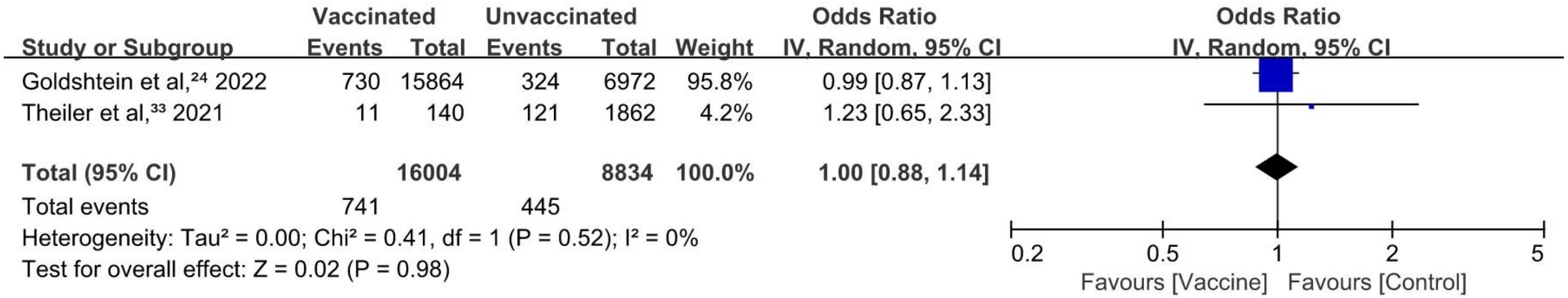

Figure S5. Forest plot for low birth weight.

Study or Subgroup	Vaccinated Mean	Vaccinated SD	Vaccinated Total	Unvaccinated Mean	Unvaccinated SD	Unvaccinated Total	Weight	Mean Difference IV, Random, 95% CI
Beharier et al,[16] 2021	3,281.8	420.2	92	3,239.4	395.7	66	1.5%	42.40 [-86.00, 170.80]
Citu(2) et al,[20] 2022	3,149	380	173	3,207	362	529	5.2%	-58.00 [-122.48, 6.48]
Conti et al,[35] 2022	2,960	510	11	3,545	583	12	0.1%	-585.00 [-1031.81, -138.19]
Dick et al,[21] 2022	3,280	451.9	2305	3,260	459.3	3313	17.2%	20.00 [-4.19, 44.19]
Fell et al,[22] 2022	3,368	526.7	22660	3,351	557.2	30115	25.2%	17.00 [7.69, 26.31]
Goldshtein et al,[23] 2021	3,200	500	7530	3,200	400	7530	22.6%	0.00 [-14.46, 14.46]
Peretz-Machluf et al,[37] 2022	3,225.5	469.4	3240	3,247.2	542.6	460	7.2%	-21.70 [-73.85, 30.45]
Rottenstreich et al,[30] 2022	3,317.8	517.8	712	3,339.5	466.3	1063	8.4%	-21.70 [-68.95, 25.55]
Wainstock et al,[34] 2021	3,224	472	913	3,227	465	3486	12.5%	-3.00 [-37.29, 31.29]
**Total (95% CI)**			**37636**			**46574**	**100.0%**	**0.81 [-15.55, 17.18]**

Heterogeneity: Tau² = 253.61; Chi² = 19.69, df = 8 (P = 0.01); I² = 59%

Test for overall effect: Z = 0.10 (P = 0.92)

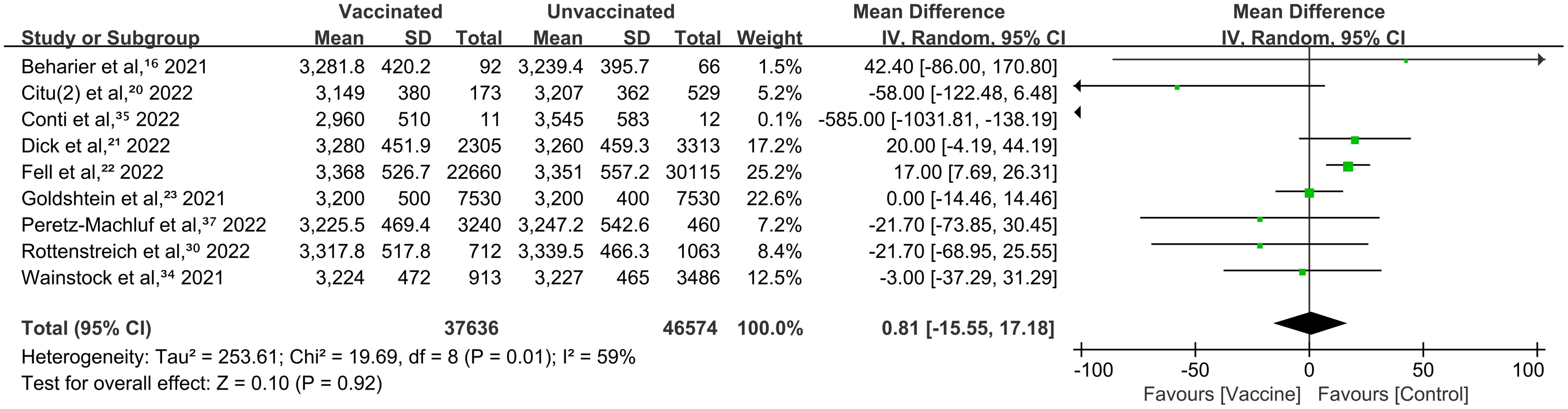

Figure S6. Forest plot for birth weight (g).

Study or Subgroup	Vaccinated Mean	Vaccinated SD	Vaccinated Total	Control Mean	Control SD	Control Total	Weight	Mean Difference IV, Random, 95% CI
Beharier et al,[16] 2021	39.3	1.3	92	39.2	1.4	66	1.8%	0.10 [-0.33, 0.53]
Conti et al,[35] 2022	38	2	11	40	2	12	0.1%	-2.00 [-3.64, -0.36]
Dick et al,[21] 2022	39.7	1.4	2305	39.7	1.3	3313	19.1%	0.00 [-0.07, 0.07]
Fell et al,[22] 2022	38.8	1.6	22660	38.8	1.8	30115	24.7%	0.00 [-0.03, 0.03]
Goldshtein et al,[23] 2021	39	0.7	1387	39	0.7	1427	22.0%	0.00 [-0.05, 0.05]
Matsui et al,[36] 2022	39.1	1.4	30	38.9	1.9	4	0.1%	0.20 [-1.73, 2.13]
Peretz-Machluf et al,[37] 2022	38.7	1.5	3240	38.8	2	460	7.3%	-0.10 [-0.29, 0.09]
Rottenstreich et al,[30] 2022	39.1	1.6	712	39.4	1.6	1063	9.9%	-0.30 [-0.45, -0.15]
Shanes et al,[32] 2021	38.5	2.4	84	38.4	1.9	116	0.9%	0.10 [-0.52, 0.72]
Wainstock et al,[34] 2021	38.9	1.4	913	39	1.9	3486	14.0%	-0.10 [-0.21, 0.01]
**Total (95% CI)**			**31434**			**40062**	**100.0%**	**-0.05 [-0.11, 0.01]**

Heterogeneity: Tau² = 0.00; Chi² = 24.14, df = 9 (P = 0.004); I² = 63%

Test for overall effect: Z = 1.64 (P = 0.10)

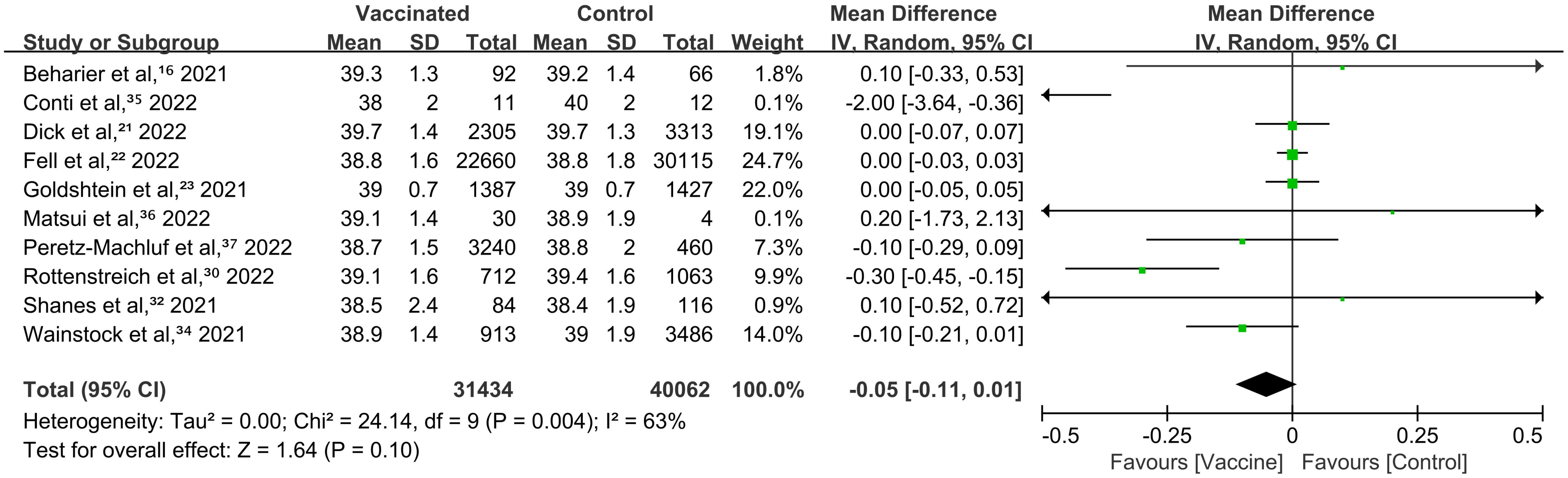

Figure S7. Forest plot for gestational age at delivery (weeks).

Study or Subgroup	Vaccinated Events	Vaccinated Total	Unvaccinated Events	Unvaccinated Total	Weight	Odds Ratio IV, Random, 95% CI
Bleicher et al,[18] 2021	2	202	1	124	0.2%	1.23 [0.11, 13.71]
Citu(1) et al,[19] 2022	124	927	271	2167	24.4%	1.08 [0.86, 1.36]
Goldshtein et al,[23] 2021	128	7530	118	7530	19.9%	1.09 [0.84, 1.40]
Magnus et al,[28] 2021	231	1003	4290	17474	55.5%	0.92 [0.79, 1.07]
**Total (95% CI)**		**9662**		**27295**	**100.0%**	**0.99 [0.88, 1.11]**
Total events	485		4680			

Heterogeneity: $Tau^2 = 0.00$; $Chi^2 = 2.03$, df = 3 (P = 0.57); $I^2 = 0\%$

Test for overall effect: Z = 0.19 (P = 0.85)

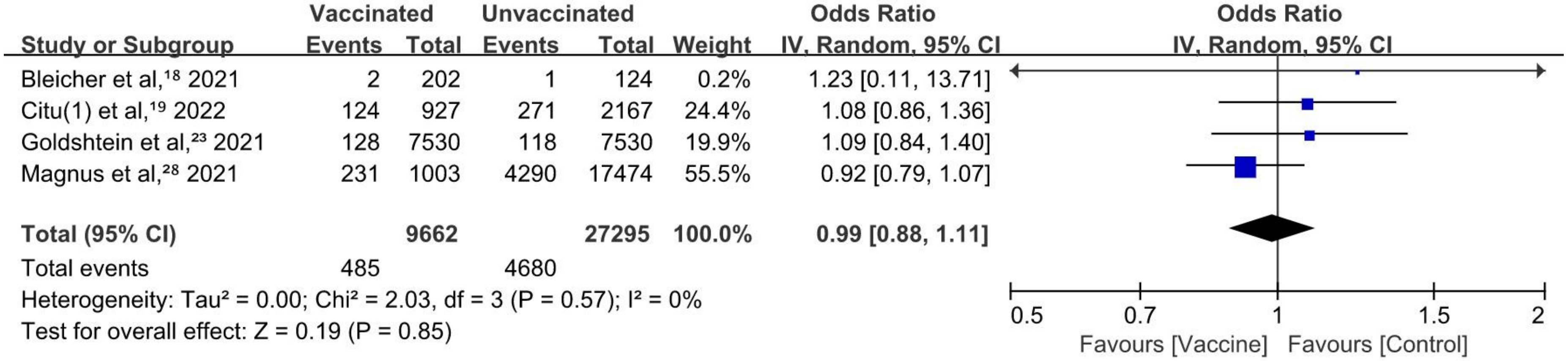

Figure S8. Forest plot for miscarriage.

Study or Subgroup	COVID-19 Vaccine Events	COVID-19 Vaccine Total	Control Events	Control Total	Weight	Odds Ratio IV, Random, 95% CI
Blakeway et al,[17] 2022	41	133	136	399	5.4%	0.86 [0.56, 1.31]
Citu(2) et al,[20] 2022	20	173	69	529	3.7%	0.87 [0.51, 1.48]
Dick et al,[21] 2022	358	2305	529	3313	18.5%	0.97 [0.84, 1.12]
Fell et al,[22] 2022	6988	22660	8583	30115	26.9%	1.12 [1.08, 1.16]
Peretz-Machluf et al,[37] 2022	1018	3686	141	458	13.6%	0.86 [0.69, 1.06]
Rottenstreich et al,[30] 2022	111	712	115	1063	9.8%	1.52 [1.15, 2.01]
Theiler et al,[33] 2021	44	140	555	1862	6.6%	1.08 [0.75, 1.56]
Wainstock et al,[34] 2021	182	913	601	3486	15.5%	1.20 [0.99, 1.44]
**Total (95% CI)**		**30722**		**41225**	**100.0%**	**1.07 [0.96, 1.19]**
Total events	8762		10729			

Heterogeneity: Tau² = 0.01; Chi² = 16.94, df = 7 (P = 0.02); I² = 59%

Test for overall effect: Z = 1.15 (P = 0.25)

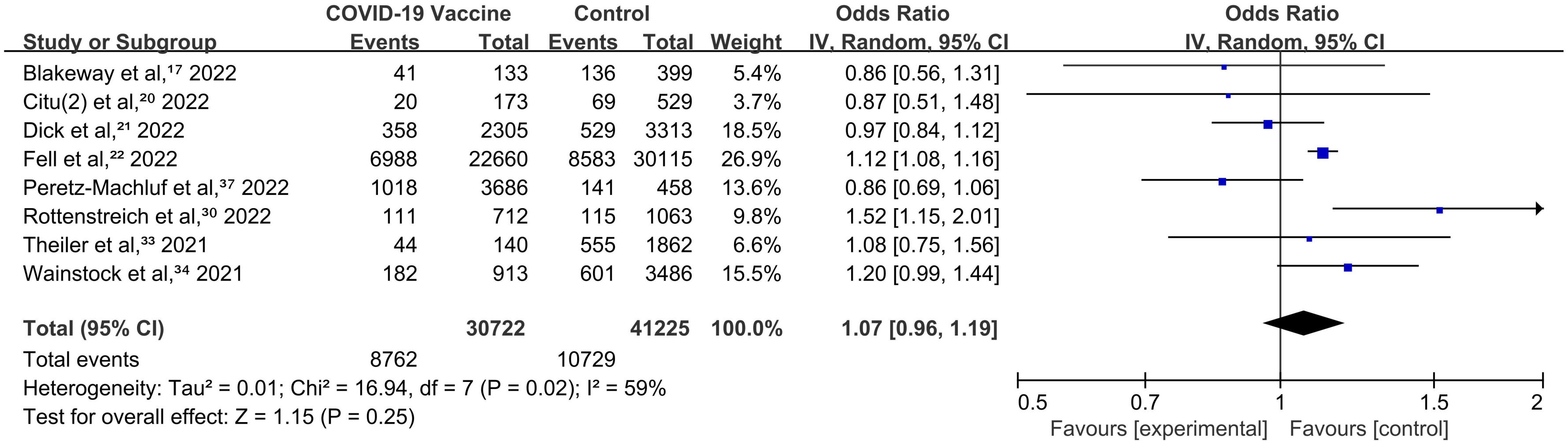

Figure S9. Forest plot for cesarean delivery.

Study or Subgroup	Vaccinated Events	Vaccinated Total	Unvaccinated Events	Unvaccinated Total	Weight	Odds Ratio IV, Random, 95% CI
Blakeway et al,[17] 2022	13	133	36	399	2.7%	1.09 [0.56, 2.13]
Citu(2) et al,[20] 2022	3	173	7	529	0.6%	1.32 [0.34, 5.15]
Dick et al,[21] 2022	79	2305	104	3313	12.6%	1.10 [0.81, 1.47]
Fell et al,[22] 2022	667	22660	1008	30115	70.6%	0.88 [0.79, 0.97]
Rottenstreich et al,[30] 2022	52	712	106	1063	9.5%	0.71 [0.50, 1.01]
Theiler et al,[33] 2021	6	140	57	1862	1.6%	1.42 [0.60, 3.35]
Wainstock et al,[34] 2021	10	913	30	3486	2.3%	1.28 [0.62, 2.62]
**Total (95% CI)**		**27036**		**40767**	**100.0%**	**0.91 [0.81, 1.01]**
Total events	830		1348			

Heterogeneity: Tau² = 0.00; Chi² = 6.32, df = 6 (P = 0.39); I² = 5%

Test for overall effect: Z = 1.77 (P = 0.08)

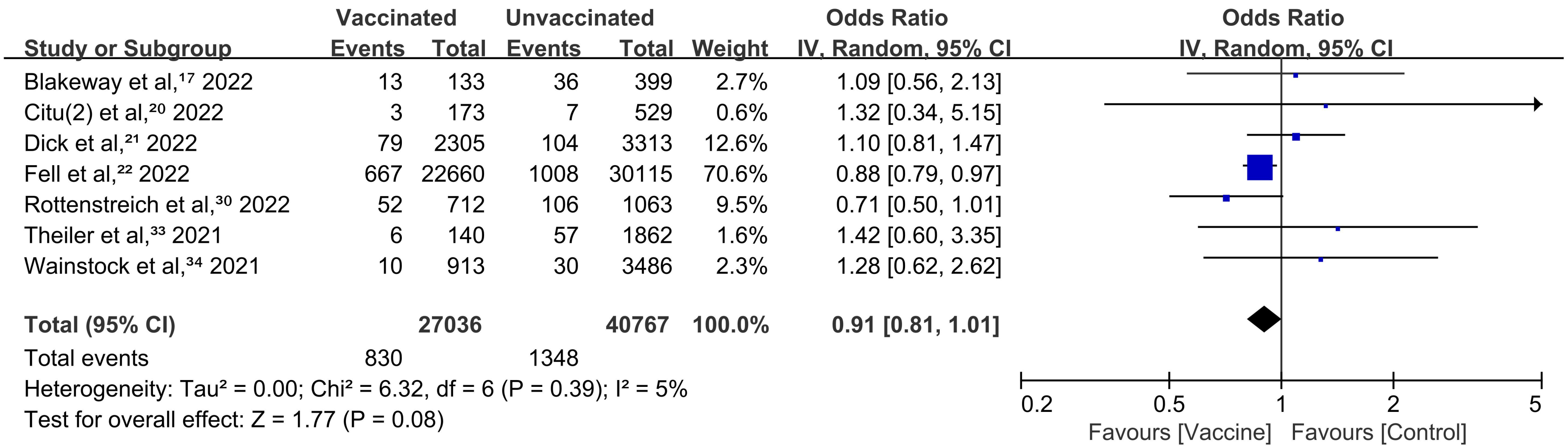

Figure S10. Forest plot for postpartum hemorrhage.

Study or Subgroup	Vaccinated Events	Vaccinated Total	Unvaccinated Events	Unvaccinated Total	Weight	Odds Ratio IV, Random, 95% CI
Fell et al,[22] 2022	101	22660	90	30115	61.7%	1.49 [1.12, 1.99]
Rottenstreich et al,[30] 2022	14	712	26	1063	38.3%	0.80 [0.41, 1.54]
**Total (95% CI)**		**23372**		**31178**	**100.0%**	**1.18 [0.65, 2.13]**
Total events	115		116			

Heterogeneity: Tau² = 0.13; Chi² = 2.92, df = 1 (P = 0.09); I² = 66%

Test for overall effect: Z = 0.53 (P = 0.59)

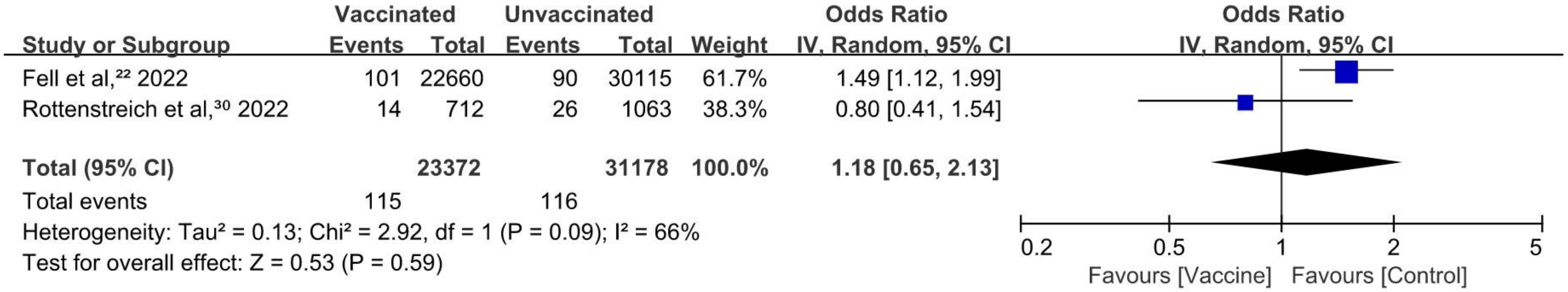

Figure S11. Forest plot for chorioamnionitis.

Study or Subgroup	Vaccinated Events	Vaccinated Total	Unvaccinated Events	Unvaccinated Total	Weight	Odds Ratio IV, Random, 95% CI
Citu(2) et al,[20] 2022	5	173	18	529	31.3%	0.84 [0.31, 2.31]
Rottenstreich et al,[30] 2022	8	712	25	1063	49.3%	0.47 [0.21, 1.05]
Wainstock et al,[34] 2021	3	913	11	3486	19.4%	1.04 [0.29, 3.74]
**Total (95% CI)**		**1798**		**5078**	**100.0%**	**0.66 [0.38, 1.16]**
Total events	16		54			

Heterogeneity: Tau² = 0.00; Chi² = 1.39, df = 2 (P = 0.50); I² = 0%
Test for overall effect: Z = 1.45 (P = 0.15)

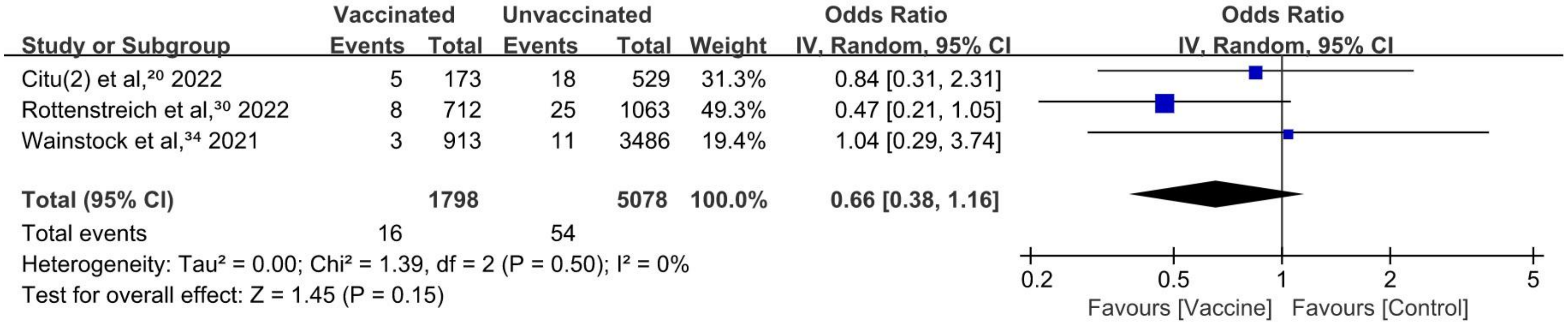

Figure S12. Forest plot for placental abruption.

Study or Subgroup	Vaccinated Events	Vaccinated Total	Unvaccinated Events	Unvaccinated Total	Weight	Odds Ratio IV, Random, 95% CI
Blakeway et al,[17] 2022	8	133	16	399	80.1%	1.53 [0.64, 3.67]
Theiler et al,[33] 2021	1	140	2	1862	19.9%	6.69 [0.60, 74.24]
**Total (95% CI)**		**273**		**2261**	**100.0%**	**2.05 [0.65, 6.50]**
Total events	9		18			

Heterogeneity: Tau² = 0.23; Chi² = 1.27, df = 1 (P = 0.26); I² = 22%
Test for overall effect: Z = 1.22 (P = 0.22)

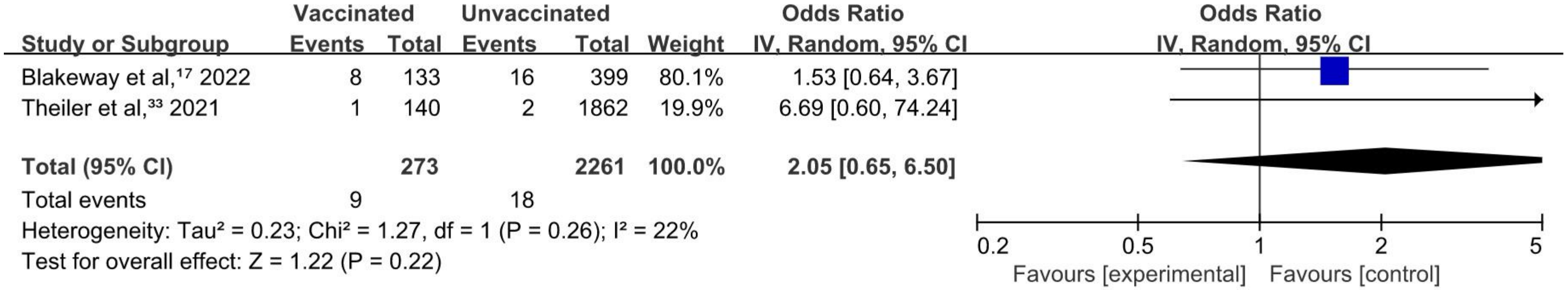

Figure S13. Forest plot for maternal ICU admission.
